# Deleterious Effects of Mycotoxin Combinations Involving Ochratoxin A

**DOI:** 10.3390/toxins5111965

**Published:** 2013-11-01

**Authors:** Maja Šegvić Klarić, Dubravka Rašić, Maja Peraica

**Affiliations:** 1 Department of Microbiology, Faculty of Pharmacy and Biochemistry, University of Zagreb, Schrottova 39, HR-10000 Zagreb, Croatia; 2 Unit of Toxicology, Institute for Medical Research and Occupational Health, Ksaverska Cesta 2, HR-10000 Zagreb, Croatia; E-Mails: dflajs@imi.hr (D.R.); mperaica@imi.hr (M.P.)

**Keywords:** mycotoxin interactions, chronic renal diseases, genotoxicity, carcinogenicity

## Abstract

Ochratoxin A (OTA) is a nephrotoxic mycotoxin with carcinogenic properties. Its presence was detected in various foodstuffs all over the world but with significantly higher frequency and concentrations in areas with endemic nephropathy (EN). Even though food is often contaminated with more than one mycotoxin, earlier studies focused on the occurrence and toxicology of only OTA. Only a limited number of surveys showed that OTA co-occurs in food with mycotoxins (citrinin-CIT, penicilic acid, fumonisin B_1_-FB_1_, aflatoxins-AF) which exert nephrotoxic, carcinogenic or carcinogen-promoting activity. This review summarises the findings on OTA and its co-occurrence with the mentioned mycotoxins in food as well as experimental data on their combined toxicity. Most of the tested mycotoxin mixtures involving OTA produced additive or synergistic effects in experimental models suggesting that these combinations represent a significant health hazard. Special attention should be given to mixtures that include carcinogenic and cancer-promoting mycotoxins.

## 1.Introduction

Mycotoxins are secondary mould metabolites and generally ubiquitous contaminants of food and feed. The mycotoxin contamination pattern is unpredictable and can be associated with several factors including weather changes, fungal crop diseases, and the ability of moulds to produce them. In terms of agricultural and animal production, the most important mycotoxins are aflatoxins-AF (B_1_, B_2_, G_1_, G_2_), ochratoxin A (OTA), fumonisins (FB_1_, FB_2_), zearalenone (ZEA) and trichothecenes (deoxynivalenol-DON, T-2, HT-2). European countries have harmonised regulations regarding levels of these mycotoxins in foods [1]. Among the listed mycotoxins, OTA deserves special attention due to several reasons: (1) OTA possesses teratogenic, embryotoxic, genotoxic, neurotoxic, immunosuppressive, carcinogenic, and nephrotoxic properties [2,3]; (2) OTA is permanently present in various foodstuffs worldwide due to the ecological variety of *Aspergillus* and *Penicillium*, both of which are OTA-producing species [4]; (3) Significantly higher OTA frequencies and concentrations in both food and human samples (urine and blood) were found in endemic nephropathy (EN) areas in Bulgaria, Croatia, and Serbia as well as in Tunisia, where a nephropathy of unknown aetiology occurred. Taking the aforementioned into account as well as findings of OTA-DNA adducts in kidneys of patients suffering from EN and the related urinary tract tumours (UTT), OTA has been suspected of being the primary aetiological agent of EN [5,6,7,8]; (4) Finally, OTA more or less frequently co-occurs with mycotoxins such as citrinin (CIT), FB_1_, penicillic acid (PA) and AF, all of which possess nephrotoxic, carcinogenic, and/or cancer promoting activity [7,8,9,10,11,12]. This article brings an overview of the recent findings on OTA occurrence in mycotoxin mixtures in foods in Europe and experimental data on the combined toxicity of OTA and CIT, PA, FB_1_ and AF.

## 2. Food Contamination with Mycotoxin Mixtures Involving OTA

Despite the fact that OTA contaminates various foodstuffs, cereals and cereal-based foods remain the primary targets of this mycotoxin. OTA is produced by several *Aspergillus* and *Penicillium* species, both of which inhabit a variety of ecological niches. *Penicillium verrucosum* is a major OTA producer in cereals, particularly in cool temperate climates in the northern hemisphere [13,14]. It can grow on grains with a moisture content of 10%–20%, while the optimal temperature for OTA production is 25 °C [15]. Apart from OTA, this species also produces CIT, but to a lesser extent when compared to OTA [16]. Aspergilli from the section *Circumdati*, which includes *A. ochraceus*, *A. westerdijkiae*, and *A. steynii*, are the most important OTA producers, often identified as *A. ochraceus* [17]. These xerophilic fungi are adapted to grain with a moisture content of 9%–16%, whereas their optimal temperature for OTA production is between 25 and 30 °C [15]. Apart from OTA, *A. westerdijkiae* and *A. steynii* are also able to produce penicillic acid [17]. *P. aurantiogriseum* produces PA and strains that are potent producers of PA have been accused of causing porcine nephropathy in Bulgaria [12,18]. Aspergilli from the section *Nigri* (*A. carbonarius* and *A. niger*) are also important sources of OTA. They contaminate tropical and dried fruits as well as grapes more frequently than cereals. Black Aspergilli also produce fumonisins (mainly FB_2_), which have until recently been attributed only to the *Fusarium* species [19,20,21].

Recent reviews [15,22] pointed towards the influence of climate changes on mycotoxin occurrence in cereals and other foods. The authors indicated that the aflatoxigenic *A. flavus* may become more problematic in temperate climates than the OTA producer *Penicillium verrucosum* if the mean temperature increases to approximately 30 °C. Also, *A. ochraceus* dominated against *A. flavus* *in situ* at 18 °C but not at 30 °C. The *Fusarium* species that produce fumonisin are considered to be field fungi mainly contributing to fumonisin content during their field growth on grains. In storage conditions and lower water activity, black Aspergilli can take up a more significant role in fumonisin as well as OTA production. Recently, *A. niger* producers of both fumonisins and OTA were isolated from maize in Portugal [23].

In the past few decades, only a limited number of mycotoxin surveys have devoted attention or specified the percent of mycotoxin co-occurrences in foods. Recent reviews [24,25] have shown that, in the last fifteen years in Croatia and the surrounding countries, only a few studies focused on mycotoxin co-occurrences in cereals. The studies undertaken in Bulgaria, Croatia, and Serbia addressed mixtures involving OTA, CIT, and FB_1_ due to their possible involvement in EN. Higher co-contaminations with OTA and CIT or OTA and FB_1_ were found in EN than in non-EN villages. These studies confirmed that EN populations were more frequently exposed to OTA and CIT due to microclimatic conditions (high humidity) and specific dietary habits. Higher levels of OTA and CIT in blood and urine, as well as higher sphinganine/sphingosine ratios (biomarker of exposure to fumonisins) in urine, confirmed that EN regions were more frequently exposed to these mycotoxins than non-EN areas [26,27,28,29]. Streit *et al.* [30] reviewed mycotoxin co-occurrences in animal feed in Europe since 2004. Since *Fusarium* species are the most frequent fungal pathogens on field crops, it was not surprising that B-trichothecenes (DON), ZEA, and FBs were the major co-contaminants. Stoev *et al.* [12] reported relatively high levels of co-occurrences of OTA (100%, 27.3–376.4 μg·kg^−1^) with FB_1_ (92%–96%, 4806–5564.1 μg·kg^−1^), CIT (92%–96%, 27.5–120.5 μg·kg^−1^) and PA (88%–92%, 86.5–904.9 μg·kg^−1^) in feed samples from pig and poultry farms in Bulgaria, where a nephropathy of unknown aetiology occurred. AFs were found in detectable levels in many European countries. Ibáñez-Vea *et al.* [31,32] reported detectable levels of AFB_1_ in 123 barley samples (Spain) and combinations AFB_1_, OTA and DON and AFB_1_, OTA, DON, and ZEA were the most frequent. The dry and hot season of 2003 in northern Italy resulted in a high incidence (75%) of AFB_1_ in maize, which led to elevated levels of AFM_1_ in cow milk, exceeding the EU limit of 0.05 μg·kg^−1^ [33]. Similarly, in 2013 AFM_1_ levels above the EU limit were found in milk in Croatia, but the Croatian Food Agency did not find AFs in concentrations above the EU limit in the feed collected from 20 feed-producing domestic companies [34] suggesting that AFs were present only in imported feed.

In the past three years, a number of papers from authors across the world dealt with the occurrence of mycotoxin mixtures in foods. The findings from European countries and the Mediterranean region from that period are summarised in Table 1. OTA was detected in all of these studies and concentrations above the EU limit were found in foodstuffs imported in Italy^b^, in spices from Turkey, and cereals from the Mediterranean (Tunisia) [31,35,36,37,38,39,40,41,42,43,44]. Most of these studies focused on the co-occurrence of OTA and AF. AF levels also exceeded the EU limit in the same foods as OTA. A recent study conducted in Croatia showed a co-occurrence of OTA, CIT, and AF in meat products [44]. Also, previous studies performed in a Croatian EN region stressed the significance of OTA intake via contaminated smoked meat [45]. Sørensen *et al*. [46] reported high levels of OTA (56–158 µg·kg^−1^) in meat products from Parma, which exceeded the maximum tolerable level (1 µg·kg^−1^) in Italy.

**Table 1:** Recent data on mycotoxin mixtures involving ochratoxin A (OTA) in foods from European countries.

Sample/No (Country)	Mycotoxin		Contamination (%)		Range (μg·L^−1^ or µg·kg^−1^)		Co-occurrence	Reference
Beer/106 (25 EU)^a^	AF		Nd		-		Two toxins	[35]
	DON		66		<0.5–18.6		41.5%	
	FB_1_		96		<0.1–30.3		Three toxins	
	FB_2_		57		<0.1–3.9		42.4%	
	OTA		66		<0.002–0.189			
Spices/105 (Turkey)	AF		21.1–79.2		0.13–37.38		4.3%–62.5%	[36]
	OTA		17.4–75 (6.7% > EU limit for both toxins)		0.06–98.2			
Cereals/110 Turkey	AF		24.5		0.022–0.233		14.6%	[37]
	OTA		43.6		0.066–1.125			
Baby milk/62 (Turkey)	AFM_1_		8		0.06–0.022		1.6%	[38]
	OTA		19,4		0.017–0.184			
Food ^b^/345 (Italy)	AF		5 (1.2% > EU limit)		0.33–70.69		Not specified	[39]
	OTA		17.6		2.76–23.7			
Pasta/27 (Italy)	AFB_1_		Nd				Not specified	[40]
	DON		81.5 (26% > EU limit)		35.1–450.0			
	OTA		96.3		0.2–0.52			
Breakfast Cereals/46 (Spain)	AF		19		0.04 ^c^		Two toxins 28%	[41]
	OTA		5		0.03			
	ZEA		43		2.87			
Barley/123 (Spain)	AF		100		0.15 ^c^		Two or three	[31]
	OTA		58		0.06		toxins 80%	
	ZEA		39		0.84			
Wheat/37 Oat bran/30 (Spain)	DON		62/17 (total of 19% > EU limit)		1308/230^c^		Two toxins 10.5% Three toxins 4.5%	[42]
	OTA		30/20		1.1/0.3			
	ZEA		13/17		8/8			
Cereals and cereal-based food/265 (Mediterranean region) ^d^	AFs		10 (8% AFB_1_ > EU limit)		4.2–66.7		Two toxins 14%> two toxins 18%	[43]
	BEA		10		2.4–844			
	DAS		2.8		6.4–97			
	DON		4.5		63.2–296			
	FB_1_		3		<LOQ-186			
	FB_2_		3.3		<LOQ-176			
	HT-2		4.5		<LOQ-87			
	NIV		50		100–903			
	OTA		1.8 (All > EU limit)		75–112			
	T-2		5		12.9–78.4			
Fermented meat/90 (Croatia)	AFB_1_		10		<1.0–3.0		Not specified	[44]
	OTA		64.4		<0.05–7.83			
	CIT		4.4		<1.0–1.0			

Notes: ^a^: 25 European countries; ^b^: Food imported in Italy (nuts, nut products, dried fruits, cereals, cereal products, pulses, dried vine fruits, coffee);^c^: Results are represented as mean concentration; ^d^: Italy, Spain, Tunisia, Morocco; Nd- not detected.

Few European countries determined maximum allowed levels of OTA in meat products: Denmark (10 μg·kg^−1^ in pig kidney), Estonia (10 μg·kg^−1^ in pig liver), Romania (5 μg·kg^−1^ in pig kidney, liver and meat) and Slovakia (5 μg·kg^−1^ in meat and milk) [44]. However, data on other important mycotoxins in meat products are lacking and the European Commission did not set regulations for such products even though this type of food is consumed in Europe on a daily basis.

## 3.Toxicity of Mycotoxin Combinations Involving OTA

The combined toxicity of mycotoxins is hard to predict based on the toxic effect of a single mycotoxin. In recent years, the number of studies on the combined toxicity of most important mycotoxins is increasing, as is the screening of mycotoxin mixtures in foodstuffs. Several reviews addressed the experimental mathematical models for the analysis of mycotoxin interactions and their combined toxicity *in vivo* and *in vitro* [25,47,48]. Grenier and Oswald [48] performed a meta-analysis of published raw data on mycotoxin interactions *in vivo* and classified the interaction into the following categories: synergistic, additive, less than additive, and antagonistic. The authors also differentiated between three types of synergistic effects and two types of antagonisms. Such characterisation of mycotoxin interactions is helpful in experimental designs and interpretations of combined toxicity outcomes and should be included into further investigations on mycotoxin interactions. 

**Figure 1:**
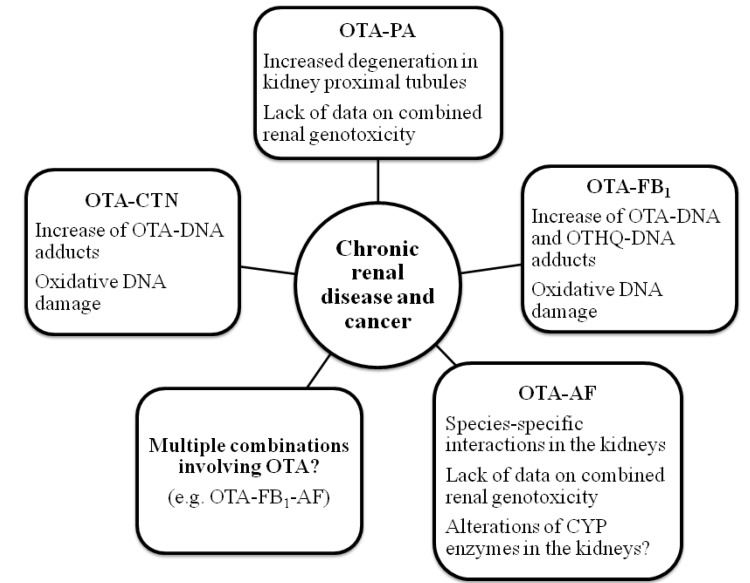
Interactions of mycotoxin combinations involving OTA and their possible role in the development of chronic renal diseases and cancer following chronic exposure to subtoxic concentrations of mycotoxin mixtures.

OTA is a nephrotoxin with potent renal carcinogenicity in animals and may be responsible for EN and urinary tract tumours (UTT) in humans [8,49,50]. The International Agency for Research on Cancer (IARC) classified OTA into Group 2B (possible human carcinogen) [3]. The mechanism of OTA genotoxicity and its role in carcinogenicity have been a controversial subject; direct genotoxic action (DNA adduct formation), indirect oxidative DNA damage, and a network of interacting epigenetic mechanisms (inhibition of protein synthesis, oxidative stress, activation of specific signalling pathways) have been proposed [51,52]. Akman *et al.* [53] showed that the oxidation of OTA by rat liver microsomes or by transition metal ions (particularly Fe(III)), as well as the hydroquinone metabolite (OTHQ) in the presence of cysteine, activates OTA and OTHQ to a directly genotoxic mutagen in human kidney cells (Ad293). Hadjeba-Medjdoub *et al.* [54] showed that the C5-Cl atom in the OTA structure is the key for the direct genotoxicity of OTA. OTA and its analogues substituted on C5 (OTBr and OTHQ) can react directly with deoxyguanosine (dG) upon photoirradiation. They can also generate covalent DNA adducts in human bronchial epithelial W126 cells and human kidney HK2 cells. Recent studies have shown that OTA induces reporter gene mutations in rat proximal tubules and that OTA alters genes encoding for regulators of DNA-double strand breaks and p53-related factors, particularly in the outer medulla [55,56]. These findings, together with the LC-MS/MS confirmation of OTA-DNA adducts [57,58], strongly speak in favour of the direct genotoxic action of OTA. As for mycotoxin mixtures that include OTA, the most important are those that involve CIT, PA, and FB_1_ due to their nephrotoxicity and possible involvement in human and animal nephropathies including EN (Table 2, Figure 1). Besides nephrotoxicity, FB_1_ acts as carcinogen as well as a promoter of carcinogenesis and could potentiate OTA genotoxicity and carcinogenicity [59,60]. AF levels in Europe are increasing [22] and their interactions with OTA, due to their carcinogenic activity, might pose a serious threat to human and animal health.

**Table 2:** *In vivo* and *in vitro* interactions of OTA with CIT, PA, FB_1_ or AFB_1_.

Experimental model	Mycotoxin combination/treatment	Effect (Interactions)	References
Opossum kidney cells	OTA + CIT (0.5–50 µM):		[76]
	concentrated OTA < concentrated CIT;	↓Cytotoxicity (AN)	
	concentrated OTA ≤ concentrated CIT	↑Cytotoxicity (A)	
	OTA (10 µM) + CIT (50 µM)	↑DNA adducts	
Human kidney cells (HK2)	OTA + CIT	↑DNA adducts ↑COX2 Inhibition of CYP 3A4 expression	[77]
Porcine urinary bladder cells	OTA (0.001–1 μM) + CIT (0.001–1 μM)/24 h	No effect on viability	[80]
V79 cells	OTA (1–10 μM) + CIT (1–10 μM)/24 h	No effect on viability	[80]
PK15 cells	OTA (6 and 10 µM)+ CIT (30 and 50 μM)/24 h	↑cytotoxicity (A)	[66]
Human proximal tubule cells	OTA (25 and 50 nm·L^−1^) + CIT (0.25 and 1 μmol·L^−1^)/24 h	No effect on caspase-3 activation	[82]
	OTA (25 and 50 nmol·L^−1^) + CIT (2.5 and 5 μmol·L^−1^)	↓caspase-3 (AN)	
	OTA (25 and 50 nmol·L^−1^) + CIT (7.5 and 15 μmol·L^−1^)	↑;caspase-3 (A)	
PK15 cells	OTA (30 and 50 μM) + CIT (6 and 10 μM)/12 and 24 h	↑apoptosis (S) ↑cytosolic calcium level ↓micronucleus rate (AN) ↑nuclear buds (A)	[66]
Vero cells	OTA (12.5 and 25 μM) + CIT (60 μM)/12 h	↓cell viability (S) ↑MDA level (S) ↑Hsp 70 expression	[63]
Rat	OTA (0.125 mg·kg^−1^ 21 d, p.o.) + CIT (20 mg·kg^−1^ p.o.)	↑plasma MDA level ↑plasma GSH level ↑hOGG1 tail intensity in liver and kidney	[83]
Rat	OTA (26 μg·kg^−1^ feed) + CIT (100 μg·kg^−1^ feed) for 21 d	↑kidney OTA-DNA adduct formation (S)	[76]
S. *typhimurium* TA102	OTA (12.3–1000.0 μg/plate) + CIT (3.0–250.0 μg/plate)	No increased mutagenicity	[77]
Chicken embryos	OTA (0.03–0.5 μg/embryo) + CIT (4 μg/embryo)	No increased teratogenicity	[85]
Pregnant rats	OTA (1 mg·kg^−1^) + CIT (30 mg·kg^−1^)/1 × s.c.	↑fetal malformations	[86]
Chicken	OTA (0.5 mg·kg^−1^ b.m. for 2 d, i.m.) + CIT (25 mg·kg^−1^ infusion)	No effect on diuresis	[87]
Broiler chicks	OTA (3.0 mg·kg^−1^ feed) + CIT (300 mg·kg^−1^ feed) 21 d	No effect on plasma constituents	[88]
Rabbits	OTA (0.75 mg·kg^−1^ feed) + CIT (15 mg·kg^−1^ feed) 60 d	↑ ultrastructural kidney changes	[89]
Dogs	OTA (0.1 and 0.2 mg·kg^−1^ b.m) p.o. + CIT (5 kg^−1^ b.m)/i.p. 14 d	Kidney necrosis Ulceration of intestinal mucosa ↑mortality	[90]
Human PBM	OTA (0.0038–12.5 mg·L^−1^) + mixture PA + CIT + FB_1_ (0.038–125 mg·L^−1^)/24 h	No effect on metabolic activity (AN)	[95]
Chickens	OTA 0.1 mg·mL^−1^ + PA 6 mg·mL^−1^/gastric intubation 20 and 28 d	↑mortality (S)	[98]
Chickens	OTA (0.13–0.8 mg·kg^−1^ feed) + PA (1–2 mg·kg^−1^ feed)/6–10 weeks	↓body weight, ↑ degenerative changes in the kidney, liver and lymphoid organs (S)	[103]
Mice	OTA (10 mg·kg^−1^ feed) + PA (40 mg·kg^−1^ feed)/10 d	↑mortality, acute multifocal toxic tubular nephrosis (S)	[99]
Pigs	OTA (0.09–0.79 mg·kg^−1^ feed) + PA (1–9 mg·kg^−1^ feed)/3–5 months	↑degenerations in kidney proximal tubules and proliferation in the interstitium (S)	[104]
Human PBM	OTA (0.0038–12.5 mg·L^−1^) + mixture CIT + FB_1_ (0.038–125 mg·L^−1^)/24 h	↑cytotoxicity (S)	[95]
C6 glioma cells, Caco-2 and Vero cells	OTA (10 µM) + FB_1_ (5, 25 and 50 µM)/24–72 h	↑cytotoxicity (S)	[116]
PK15 cells	OTA (0.05, 0.5 and 5 µg·mL^−^^1^) + FB_1_ (0.05, 0.5 and 5 µg·mL^−^^1^)/24 h	↑cytotoxicity, lipid peroxidation and micronuclei (AD); ↓ glutathione level (AD), ↑caspase-3 (S)	[117,118,121]
Turkey poults	OTA (3 mg·kg^−1^ feed) + FB_1_ (300 mg·kg^−1^ feed)/3 weeks	↓body weight, ↑AST, ALT, creatinine (S); serum triglycerides (AN)	[119]
Rabbits	OTA (2 mg·kg^−1^ feed) + FB_1_ (10 mg·kg^−1^ feed)/45 d	↑ALP (AD); ↑AST, ALT (LAD)	[120]
Rats	OTA (5 ng·kg^−1^, 0.05, 0.5 mg·kg^−1^) + FB_1_ (200 ng·kg^−1^, 0.05, 0.5 mg·kg^−1^)/p.o. 15 d	↑lipid peroxidation, protein carbonyls, DNA damage in the kidneys (S); ↓ catalase (S); ↑DNA adducts (S)	[8,112,113]
Pigs	OTA (0.5 mg·kg^−1^ feed) + FB_1_ (10 mg·kg^−1^ feed)/35–49 d	↑kidney damage, AST, ALT, creatinine (S); ↓ antibody titer against the Morbus Aujeszky (S)	[122]
Vero cells	OTA + AFB_1_ (5–50 µM)/24 hOTA (1 µM) + AFB_1_ (1–20 µM)OTA (1–20 µM) + AFB_1_ (1 µM)	↑cytotoxicity (A) ↑DNA damage, p53 ↓bcl-2	[129]
HepG2 cells	OTA 1–200 µM + AFB_1_ (100 or 150 µM)/24 h	↑cytotoxicity (AD); ↓ DNA damage (AN)	[130]
Rats	Single doses OTA 0.5 mg·kg^−1^ + AFB_1_ 0.25 mg·kg^−1^ p.o.	No interactions in acute liver toxicity, kidney or immunological organs damage	[133]

Notes: COX2: cycloxygenase; PMB: peripheral blood mononuclear cells; AST: aspartate aminotransferase; ALT: alanine aminotransferase; ALP: alkaline phosphatase; S: synergism; AD: additive; LAD: less than additive; AN: antagonism.

### 3.1.Combined Toxicity of OTA and CIT

CIT is a primarily nephrotoxic mycotoxin produced by various species of *Penicillium*, *Aspergillus*, and *Monascus*. Ten years after its isolation from *Penicillium citrinum*, its antibiotic properties were discovered but never used due to its nephrotoxicity [61,62]. The mechanism of CIT toxicity was studied exclusively *in vitro* and has not been completely elucidated. It seems that several mechanisms are involved, such as lipid peroxidation, alteration of mitochondrial function by disturbing Ca homeostasis, and induction of apoptosis by the activation of caspase-3, -6, -7 and -9 [63,64,65,66,67]. In contrast to its evident nephrotoxicity, CIT genotoxicity studies did not yield unequivocal results. CIT induced chromosomal aberrations in the bone marrow of treated mice [68], DNA single strain breaks in *E. coli* [69] and increased number of micronucleus in PK15 cells [66], HepG2 cells [70], human lymphocytes [71], and V79 cells [72]. However, the increased frequency of sister chromatid exchange was not found in V79-E cells, human lymphocytes, and CHO-K1 (Chinese hamster ovary cells) [73,74]. The significant change in tail moment values of the Fpg comet assay was not noticed in CIT-treated HEK293 (human embryonic kidney cells) but the tail intensity in rat kidney cells increased when analysed with the hOGG1 comet assay [74,75]. However, CIT-DNA adduct formation was detected either in human cultured cells or in rat kidneys [76,77]. CIT was not mutagen when tested on *Salmonella typhymurium* TA 102 [78]. IARC evaluated the carcinogenic properties of CIT and classified it into Group 3 (non-carcinogen to humans) [79].

Simultaneous treatment or exposure of cells and experimental animals to OTA and CIT resulted in antagonistic, additive, and synergistic effects, which depended on the applied doses, type of tested cells, and the observed endpoint. No change in cytotoxicity was reported on porcine urinary bladder epithelial cells (PUBEC) and V79 cells when treated simultaneously with OTA and CIT for 24 hours and compared with single mycotoxin-treated cells [80]. Šegvić Klarić *et al.* [66] found an additive cytotoxic effect of the simultaneous administration of OTA and CIT on PK15 cells in contrast to a study by Heusner *et al.* [81], where the treatment of another porcine renal cell line (LLC-PK1) resulted in synergism. Caspase-3 was not activated in immortalized human proximal tubule cells (IHKE) after a 24-hour treatment with 25 and 50 nM OTA and 0.25 and 1 µM of CIT [82]. When the CIT concentrations were increased (2.5 and 5 µM), the effect was antagonistic, while a further increase of CIT concentrations (7.5 and 15 µM) gave an additive effect. When the apoptotic effect of combined treatment of PK15 cells with OTA (30 and 50 µM) and CIT (6 and 10 µM) was studied, the synergism was dose dependent and more pronounced after 12 h than after 24 h of treatment [66]. However, the same treatment had an antagonistic effect on the micronucleus rate and an additive increase of the number of nuclear buds. The combined effect of these two mycotoxins may cause oxidative stress because there are reports on the significantly increased malondialdehyde (MDA) concentrations either in cell cultures or in kidney and liver of treated rats as compared to single mycotoxin treatments [63,83]. In a more recent study, both mycotoxins caused an increase in the tail length and tail intensity of kidney and liver cells as measured with the hOGG1 comet assay. These results are in accordance with the DNA fragmentation found in Vero cells within another study [84]. The genotoxic properties of the combination of these two mycotoxins were further confirmed by a 10-fold increase of a major OTA-DNA adduct in rat kidneys [76]. A mutagenicity study involving combined treatment with OTA and CIT did not induce reverse mutations in *Salmonella typhimurium* strain TA102 probably because of the antibiotic properties of CIT [78]. The appearance and severity of malformations was not significantly increased in the single teratogenicity study performed on chicken embryos exposed to OTA and CIT [85]. However, in dams of OTA- and CIT-treated pregnant rats gross malformations, visceral anomalies and skeletal defects increased significantly depending on the day of gestation when treated [86]. It seems that the effect of the simultaneous treatment of experimental animals with OTA and CIT is species-related. In the two studies on chicks and broilers, such treatment did not cause an increase of diuresis or renal tissue damage [87,88]. In contrast, the simultaneous exposure of mammals to both mycotoxins significantly increased kidney lesions, which were most dominant in the proximal convoluted tubules [89,90].Ultrastructural renal alterations in New Zealand white rabbits treated with OTA (0.75 mg·kg^−1^ feed) and CIT (15 mg·kg^−1^ feed) alone or simultaneously for 60 days revealed more severe lesions than in the individual toxin-treated groups [89].In the group of animals treated with both mycotoxins, renal changes were seen in all parts of the kidney, but were most prominent in the mitochondria of proximal convoluted tubules, where the basement membrane of the glomeruli thickened and the endothelial cells degenerated. 

### 3.2.Combined Toxicity of OTA and PA

PA is a lactone substance that was first isolated from *Penicillium puberulum* by Alsberg and Black in 1913 [91]. Since then, it has been detected in different *Penicillium* and *Aspergillus* species. As many lactones, PA possess a wide range of toxic properties including carcinogenicity. Dickens and Jones [92] observed tumours in mice subcutaneously injected with 1 mg PA twice a week for 64 weeks [91]. Umeda *et al.* [93] showed that PA induces DNA-strand breaks in HeLa cells. However, IARC classified PA as belonging to Group 3, which means that PA is not carcinogen to humans [94]. In human peripheral blood, mononuclear cells (PBM) PA and OTA exert an opposite effect on metabolic activity as measured by an MTT test; PA increased while OTA decreased metabolic activity, whereas a combination of OTA + PA did not produce any kind of synergism [95]. Studies on experimental animals revealed that PA (90 mg·kg^−1^) induces significant hepatobiliary excretory dysfunction in mouse and rats, while it did not provoke significant damage to internal organs in chickens upon administering up to 400 mg·kg^−1^ [96,97]. In chickens and mice, OTA and PA showed synergism in the increase of mortality [98,99]. More pronounced damage of proximal tubules in the kidney was observed in mice [99].PA inhibits carboxypeptidase—the enzyme involved in the detoxification of OTA—which might enhance OTA toxicity [100].

*Stoev et al.* [12,101] suggested that Bulgarian and South African porcine/chicken nephropathy may have a multitoxic aetiology because OTA concentrations in animal feed were not high enough to induce nephropathy [12,18,101,102]. This theory was supported by several studies: (1) Both OTA and PA were detected in feed in Bulgaria where nephropathy occurs and PA was present in two to three times higher concentrations than OTA; (2) Besides OTA and PA, feed was also contaminated with high levels of FB_1_ and penitrem A, and low levels of CIT, DON, and ZEA. Contamination with PA and FB_1_ was above 88%, suggesting that both PA and FB_1_ might contribute to nephrotoxicity [12]; (3) Experimentally induced nephropathy in pigs and chickens fed with OTA and PA in concentrations that were naturally present in Bulgarian feed samples revealed similar degenerative changes in the kidneys as was seen in spontaneous cases of nephropathy [18,102]; (4) When simultaneously treated with these toxins, synergistic interactions between OTA and PA were recorded in pigs and chicks [103,104,105]. Taking into account the carcinogenic activity of PA reported by Dickens and Jones [92] and proven OTA carcinogenic properties, this combination might have higher carcinogenic potential than single toxins.

### 3.3.Combined Toxicity of OTA and FB_1_

Leukoencephalomalacia in horses and porcine pulmonary oedema were the first acute FB_1_-induced toxicoses described in farm animals [106]. Studies on rodents revealed that FB_1_ possesses gender-specific hepatotoxic, nephrotoxic and carcinogenic properties. FB_1_ inhibits ceramid synthase, a key enzyme of the sphingolipid metabolism, which in turn affects various signalling pathways within cells [106]. An NTP study [107] showed that FB_1_ induces nephrotoxicity and renal carcinogenicity in rodents. FB_1_ was also believed to be the cause for the development of human oesophageal cancer in South Africa and China [108]. IARC classified FB_1_ in Group 2B as a possible carcinogen to humans [59]. However, its carcinogenicity mechanism is still unknown. Studies on FB_1_-induced genotoxicity are inconsistent. Negative results were obtained for gene mutation and DNA repair tests in *E. coli* as well as unscheduled DNA synthesis in rat hepatocytes [109,110,111]. By contrary, FB_1_ caused an increase in micronuclei formation in different cell lines and DNA strand breaks in rat liver and kidney as measured by alkaline and Fpg-modified comet assay [112,113]. The last one implicated oxidative stress in FB_1_-mediated genotoxicity [113]. Studies taken so far suggested that FB_1_-disruption of sphingolipid signalling pathways which control cell growth, motility, vascular barrier integrity, and angiogenesis play a key role in tumour formation rather than direct genotoxic action [106,114,115].

The combined effects of OTA and FB_1_ were intensively studied over the last decade due to their frequent co-occurrence in foodstuffs and nephrotoxic and carcinogenic properties. In both *in vitro* and *in vivo* studies, these toxins interacted in a synergistic or additive manner [25]. Creppy *et al.* [116] reported cytotoxic synergism between low FB_1_ and high OTA concentrations in rat brain glioma C6 cells, human intestinal Caco-2 cells, and Vero cells. In human PBM, mixtures of FB_1_ and CIT with OTA also showed synergistic cytotoxicity [95]. Subcytotoxic concentration of OTA and FB_1_ additively increased lipid peroxidation and decreased the level of glutathione in PK15 cells while inducing caspase-3 in a synergistic manner [117,118]. These *in vitro* studies indicate that the type of interaction between OTA and FB_1_ depends on the concentrations that will induce oxidative stress as well as impair protein synthesis. Some studies *in vivo* on turkey poults, rabbits, and rats also showed that the type of interaction between OTA and FB_1_ is influenced by the dosage. In turkey poults, the combination exerted synergism in the reduction of body weight, biochemical parameters, and enzyme levels when a high FB_1_ concentration was applied [119]. By contrary, when low doses of toxins were used in rabbits, additive or less than additive interactions on biochemical parameters and enzyme levels were obtained [120]. Using doses that correspond to the human daily intake of FB_1_ and OTA, Domijan *et al.* [112] observed a synergistic effect on the parameters of oxidative stress in rat liver and kidneys. Regarding the genotoxicity of the mixture, a dominant additive genotoxic effect in PK15 cells was obtained with a micronucleus assay, which detects fixed mutations, showing that both OTA and FB_1_ have genotoxic potential [121]. The genotoxicity of OTA and FB_1_, as well as that of their mixture, was confirmed in rats and pigs [60,113]. In rats intraperitoneally treated with OTA and FB_1_ doses that reflect the daily intake of these toxins in Europe, Fpg-modified and standard alkaline comet assay showed that the combination synergistically induced DNA damage in the animal kidneys [113]. In rats and pigs fed with a mixture of OTA and FB_1_, an increase in the number of OTA-specific DNA adducts including C-C8dG OTA adduct and both OTHQ-related adduct was observed, suggesting that FB_1_ promotes OTA genotoxicity. These specific adducts are found in human urothelial tumours in EN regions [8,60]. The possible multi-toxin aetiology of EN and porcine nephropathy in Bulgaria and South Africa are supported by experiments from Stoev *et al.* [12,101,122] on pigs: the combination of OTA and FB_1_ caused stronger lesions in the kidneys, more pronounced changes in biochemical parameters, and disturbances in the humeral immune response in doses that correspond to those found in cases of porcine nephropathy in Bulgaria and South Africa.

### 3.4.Combined Toxicity of OTA and AF

Aflatoxins (B_1_, B_2_, G_1_, G_2_) are a group of potent hepatotoxins and carcinogens mainly produced by *Aspergillus flavus* and *A. parasiticus*. They have been responsible for several acute aflatoxicosis outbreaks in humans in Southeast Asia and Africa as well as the hepatocellular carcinoma prevalent in China [123,124,125]. Among AF, AFB_1_ is the most prevalent and most toxic metabolite. Its toxicity and carcinogenicity are linked to the metabolic conversion by the liver cytochrome P450 monooxygenase (CYP1A2 and CYP3A4) into the electrophilic intermediate AFB_1_-8,9-exo-epoxide which binds to DNA, RNA, and proteins [123]. Monooxygenases are also involved in the biotransformation of AFB_1_ into AFM_1_, which is secreted into the milk. AFM_1_ can also undergo epoxidation to form AFM_1_-8,9-epoxide that binds to DNA [123,124]. Therefore, IARC classified AF as Group I carcinogens [126]. CYP1A2 monooxygenase is active when AFB_1_ is present in low concentrations usually found in food, whereas CYP3A4 contributes to epoxidation at relatively high substrate concentrations [127]. Eaton and Gallagher [124] pointed out that the genetic variability in the expression of cytochrome P450 might contribute to individual differences in the susceptibility to the carcinogenic effects of AF. Even though the liver is the primary site of AF biotransformation and toxicity, kidneys also take part in the detoxification of AF and their residues have been detected in these organs [128].In cultured monkey kidney Vero cells, the combination of AFB_1_ and OTA caused additive interactions with regard to a decrease in cell viability, increased DNA fragmentation and p53 activation and decreased expression of the antiapoptotic factor bcl-2 [129]. It was proposed that AFB_1_ might yield oxidative stress due to the induction of expression of heat shock protein-70, increase of lipid peroxidation, decrease of cell antioxidants, and formation of deoxyguanosine adducts in rat liver [123]. Therefore, the potential role of oxidative stress in AFB_1_ and OTA combined genotoxicity was tested by the Fpg-modified comet assay in hepatocellular carcinoma epithelial cells (HepG2). Interestingly, the combination provoked a significant decrease in DNA damage, as compared to treatment with AFB_1_ alone. At the same time, a dichlorofluorescein assay showed that ROS levels increased. The authors put forward a hypothesis that AFB_1_ and OTA compete for the same CYP enzymes that represent the bioactivation route for AFB_1_, which in turn yields more ROS and less AFB_1_-DNA adducts [130].

Most studies addressing the toxicity of mycotoxin mixtures in animals encompass combinations involving AF. Interactions of AF with FB_1_, OTA or T-2 toxin were the most studied *in vivo*. Grenier and Oswald [48] made a meta-analysis of 17 reports on AF and OTA interactions in animals including chickens (11 reports), laying hens (two reports), pigs (two reports), calves (one report), and Guinea pigs (one report). The observed effects varied between synergistic and antagonistic, depending on the doses that were used. Briefly stated, the synergistic and additive interactions were obtained for an increase of mortality in chickens, increased number of abnormalities in chicken embryos, decrease of egg production in laying hens, decrease of feed intake, body weight and relative weight of internal organs in most animals, atrophy of lymphoid organs, and suppression of cell-mediated immunity in chickens. By contrast, less than additive and antagonistic interactions dominated the biochemical parameters, including serum concentrations of cholesterol, albumin, total proteins, creatinine, uric acid, and blood urea nitrogen [48]. The data on microscopic lesions in the liver and kidneys of chickens and pigs fed with AF-OTA mixture are inconsistent and species-specific. Huff and Doerr [131] reported that ochratoxin A inhibited lipid accumulation in the liver of chickens, which is normally induced by aflatoxins. At the same time, the primary effect of this interaction was nephrotoxicity but not hepatotoxicity. Shakare *et al.* [132] observed more severe pathological changes in both the liver and kidneys of broilers fed co-contaminated feed than animals treated with a single toxin. Injuries in the kidney tubular epithelium of animals receiving single toxins appeared before the degenerative changes in the liver. In pigs, a combination AF + OTA did not induce more prominent hepatic lesions than only AF, while in the kidneys it induced less severe lesions upon treatment with both toxins than OTA alone, thus indicating antagonism [48]. Recently, simultaneous oral administration of single doses of AFB_1_ (0.25 mg·kg^−1^ bw) and OTA (0.5 mg·kg^−1^ bw) in rats induced acute liver toxicity, which was attributed to a single AFB_1_. At the same time, no remarkable toxicity was observed in the kidneys or immunological organs. Interestingly, AFB_1_ and its metabolites disappeared within 24 h which suggested that OTA somehow accelerated the AFB_1_ metabolism and excretion, while plasma and tissue levels of OTA were not affected by AFB_1_ [133]. In most of the aforementioned studies, AFB_1_ and OTA concentrations in feed were between 0.2–3.5 mg·kg^−1^ and 0.2–4 mg·kg^−1^, respectively. These concentrations caused acute toxicity and are some orders of magnitude higher than those naturally occurring. So far there have been no studies focused on the long-term exposure of animals to the mixture of AFB_1_ and OTA in low concentrations naturally found in foodstuffs. Taking into account recent studies on rats and HepG2 cells [130,133], the genotoxicity mechanism of AFB_1_-OTA combination should be further explored with regard to the effects of naturally occurring toxin concentrations on enzymes involved in toxin metabolisms in the liver and kidney cells (Figure 1).

## 4. Conclusions

In the forthcoming period, climate change will affect agricultural practice and the ecological niches of mycotoxigenic fungi in a particular area. Mycotoxin producers in temperate climates will be replaced by better adapted species or mutants which may produce new secondary metabolites. In any case, mycotoxins will continue to occur in mixtures rather than alone, but some toxins could “overpower” others (e.g., aflatoxins). In the past few years, many mycotoxin surveys in European and Mediterranean countries were aimed at the co-occurrence of mycotoxins in foodstuffs, particularly cereals and cereal-based products. OTA was detected in most of these surveys, sometimes in concentrations above the EU limit, and in mixtures with AF and *Fusarium* toxins. Besides cereals and cereal-based food, meat products can also be significant sources of OTA and *Penicillium* toxins such as CIT or PA. Apart from regulations or recommendation regarding OTA in meat products set by few European countries, data on other mycotoxins intake through meat are lacking, even though meat products are consumed in Europe on a daily basis. Most of the studies addressing the effects of OTA-CIT, OTA-PA, OTA-FB_1_, and OTA-AF combinations have shown additive or synergistic interactions. However, less than additive and antagonistic interactions were also observed particularly for the OTA-AF combination. These discrepancies could be related to the quality of the experimental model, duration of exposure, concentrations of toxins, and the endpoints that were studied. The majority of studies on mycotoxin mixture effects employed combinations of two toxins. Mycotoxin surveys of foodstuffs showed that OTA co-occurs with more than one mycotoxin and its naturally occurring concentrations are far less than those causing acute toxicity. Therefore, future studies on *in vitro* and *in vivo* models should include several vital steps: (1) multiple mycotoxin mixtures and concentrations that naturally occur in foods; (2) mathematical/statistical design for classification of interactions into synergistic, additive, less than additive, and antagonistic; and (3) endpoints regarding genotoxicity and carcinogenicity of the mixtures (e.g., OTA-AF, OTA-FB_1_, OTA-CIT or OTA-PA). Regulatory guidelines for mycotoxins in foodstuffs should take into consideration the results of such investigations.
